# The Sig27 multigene stratifies breast cancer fatality risk via reflecting tumor-associated immune suppressive features

**DOI:** 10.1016/j.tranon.2026.102820

**Published:** 2026-05-18

**Authors:** Yingying Su, Ying Dong, Tao Zhang, Damu Tang

**Affiliations:** a Urological Cancer Center for Research and Innovation (UCCRI), St Joseph’s Hospital, Hamilton, ON L8N 4A6, Canada; b Department of Medicine, McMaster University, Hamilton, ON L8S 4K1, Canada; c The Research Institute of St Joe’s Hamilton, St Joseph’s Hospital, Hamilton, ON L8N 4A6, Canada; d Key Laboratory of Microecology-Immunomodulatory Network and Related Diseases, School of Basic Medicine, Jiamusi University, Jiamusi, Heilongjiang 154007, China

**Keywords:** Breast cancer, Biomarker, Immunosuppressive cells, Immune checkpoint, Prognostic prediction, Overall survival

## Abstract

We evaluated the biomarker potential of Sig27, a 27-gene panel originating from prostate cancer, in breast cancer (BC). Orthotopic BC tumors were generated in Balb/c mice using 4T1 cells expressing either empty vector (EV) or the gain-of-function PCSK9 mutant D374Y. Sig27 expression was analyzed in mouse tumors, 13,103 primary BCs, 60 metastases, and 780 normal breast tissues. Its prognostic value was assessed in BC, 20 other cancer types, and in tumor-associated macrophages (TAMs), regulatory T cells (Tregs), and exhausted CD8⁺ T cells (CD8Tex). Sig27 was significantly altered in D374Y vs. EV tumors and showed consistent dysregulation in primary and metastatic BCs. It stratified BC fatality risk comparably to OncotypeDX and MammaPrint. Notably, Sig27 predicted poor prognosis across 21 cancer types. In 2716 BCs, OncotypeDX and MammaPrint correlated with mitotic progression, while Sig27 was predominantly associated with immune regulation. In bulk RNA-seq datasets (*n* = 6734), Sig27 and its key genes (FPR3, LAMP3, and FAM65B) were strongly associated with multiple immune checkpoints (ICs). Single-cell RNA-seq data revealed their primary expression in TAMs, and to a lesser extent in Tregs and CD8Tex. These associations were enhanced in Sig27IM, formed by FPR3, LAMP3, and FAM65B. Their expression, along with *Pd-l1, Lgals9*, and *Pvrig*, was upregulated in D374Y tumors. Furthermore, Sig27 and Sig27IM predicted pathologic complete response in BC treated with PD1 and PD-L1 inhibitors; Sig27IM displayed robust correlations with established immune-signatures: CTL, Merck18, TIDE, and STAT1_sig. Collectively, Sig27 captures BC’s immune properties, which likely contribute to its biomarker potential.

## Highlights

• Sig27 predicts breast cancer prognosis as effective as Oncotype DX and Mammaprint.
• Sig27 effectively assesses poor prognosis across 24 human cancer types.
• Sig27 strongly associates with immune checkpoints (IC), TAM, Treg, and CD8Tex.
• FPR3, LAMP3, and FAM65B correlate with breast cancer’s immunosuppressive features.
• FPR3, LAMP3, FAM65B, CD274, TIGIT, and PVRIG are increased in advanced breast cancer.

## Introduction

Breast cancer (BC) accounted for 2.3 million new cases in 2022, representing 11.6 % of all cancer diagnoses worldwide [1]. Histologically, BC is categorized into three major classes: estrogen and/or progesterone receptor-positive (ER+, ∼70 %), HER2-positive (HER2+, 15–20 %), and triple-negative (TN, ∼15 %) [2]. At the gene expression level, BC comprises six intrinsic subtypes: luminal A and B (ER+), normal-like, HER2-enriched, basal-like, and claudin-low, with the latter two typically triple-negative [3,4]. These classifications offer valuable prognostic and therapeutic guidance. However, BC remains the leading cause of cancer-related death among women, with an estimated 670,000 deaths globally in 2022 [1] (WHO data accessed June 16, 2025), highlighting the need for improved prognostic tools.

For early-stage ER+, HER2-negative, and node-negative (or ≤3 positive nodes) BCs, several multigene panels, including OncotypeDX, MammaPrint, EndoPredict, and Prosigna, have been developed to predict recurrence risk [[5], [6], [7]]. Among these, OncotypeDX [8,9] and MammaPrint [10] have been validated in prospective clinical trials. Notably, these panels primarily assess genes involved in cell cycle progression [11,12], suggesting their prognostic value is tied to tumor proliferation. In contrast, immune evasion, another hallmark of cancer, remains underexplored as a basis for BC risk assessment [13].

To address this gap, we evaluated Sig27, a 27-gene panel originally identified in prostate cancer [14], for its prognostic potential in BC. Sig27 stratifies BC fatality risk comparably to OncotypeDX and MammaPrint. However, unlike these proliferation-based panels, Sig27 is strongly associated with immune regulation. In multiple BC datasets, Sig27 and its key component genes (FPR3, LAMP3, and FAM65B) are primarily expressed in tumor-associated macrophages (TAMs) and robustly correlate with immune checkpoints, including PD-L1, LGALS9, and PVRIG. In murine models, the upregulation of mouse homologs (Fpr1/2, Lamp3, Fam65b) and immune checkpoints (Cd274, Lgals9, Pvrig) was observed in aggressive 4T1 tumors, supporting the immunological relevance of Sig27.

Together, these findings suggest that Sig27 stratifies BC prognosis partially via its association with tumor immunosuppressive features. Its pan-cancer utility, evidenced by predicting poor outcomes with AUC ≥ 0.6 across 20 cancer types in approximately 7000 patients, further indicates its translational potential. Given its origin in prostate cancer, the application of Sig27 in BC offers a unique and promising avenue for clinical use.

## Materials and methods

### Formation of orthotopic breast cancer in syngeneic mice

Murine 4T1 breast cancer cells (ATCC) were cultured in RPMI-1640 medium supplemented with 10 % fetal bovine serum (Gibco) and 1 % penicillin-streptomycin (Gibco). Mycoplasma contamination was routinely tested using a PCR-based detection kit (Abm, Cat#: G238). Stable expression of either empty vector (EV) or the gain-of-function PCSK9 mutant (D374Y) in 4T1 cells was established via retroviral transduction [15]. For in vivo studies, 2 × 10⁴ 4T1 EV or 4T1 D374Y cells were orthotopically implanted into the mammary fat pad of Balb/c mice (*n* = 6 per group). Tumor growth was monitored weekly by caliper measurement, and volume calculated using the formula: *V* = *L* × *W*² × 0.52. Experimental endpoints were defined as tumor volume reaching ≥1000 mm³. All animal procedures were approved by the McMaster University Animal Research Ethics Board (AUP#: 24-04).

### Western blot, hematoxylin and eosin staining (H&E), and immunohistochemistry (IHC) analyses

These routine analyses were performed as we have published [16]. The antibodies utilized are presented in Supplementary Table S1. Quantification of IHC staining of PCSK9 in 4T1 tumors was performed using ImageJ.

### Semi-quantitative real-time PCR

Total RNA was isolated from 4T1 EV and 4T1 D374Y allografts using Direct-zol RNA MiniPrep Plus (Zymo Research, Cat #: R2070). Reverse transcription was performed using qScript cDNA synthesis kit (Quantabio, Cat#:95047). Semi-quantitative real-time PCR was conducted with the QuantStudio3 Fast Real-Time PCR System (Applied Biosystems, Foster, California, USA) using SYBR-green (Applied Biosystems, Cat#:4367659). Fold alterations were calculated using the formula: 2^−ΔΔCt^. The relevant real-time PCR primers are presented in Supplementary Table S2.

### Programs and websites

This study used the following programs: R2: Genomics Analysis and Visualization Platform (http://r2.amc.nl http://r2platform.com), TISCH2 [17], Metascape [18], and CZ CELLxGENE Discovery [19]. The R *glmnet, survival, Maxstat*, and other packages were also utilized.

### Data sources

Fifty-two datasets were used, including bulk RNA-seq and single-cell RNA-seq (scRNA-seq) datasets for breast cancer and other cancer types for a total of 81,211 patients. The dataset details are presented in Supplementary Table S3.

### Calculation of multigene risk scores for individual tumors

Risk scores for Sig27, Oncotype DX, and MammaPrint were yielded by obtaining coefficient (coef) of Sig27, Oncotype DX, and MammaPrint component genes in predicting overall survival (OS) probability using multivariate Cox pH regression within the R *Survival* package. Risk scores were then calculated for individual tumors with the formula: Sum (coef_1_ x Gene_1exp_ + coef_2_ x Gene_2exp_ + … …+ coef_n_ x Gene_nexp_), where coef_1_ … coef_n_ are the coefs of individual genes and Gene_1exp_ … … Gene_nexp_ are the expression of individual genes.

### Sensitivity analyses of Sig27 across BC datasets

We performed across-cohort harmonization in three independent BC datasets (TCGA, Metabric, Gruvberger-Sall-3207-tpm-gse202203) using YuGene [20], trained on each dataset, and tested the other two cohorts with fixed coefficient. Hazard ratio (HR) per SD (standard deviation) and 95 % CI (confidence interval) were computed.

### Statistical analysis

Kaplan-Meier curves, the log-rank test, and Cox regressions were conducted using the R Survival package along with tools from R2 and cBioPortal. Additional statistical analyses were carried out using various web-based applications and GraphPad Prism 7. Data is expressed as mean±SEM/SD, with a *p*-value of <0.05 deemed statistically significant.

## Results

### Sig27 is novel and relevant to BC progression

**Fig. 1:**
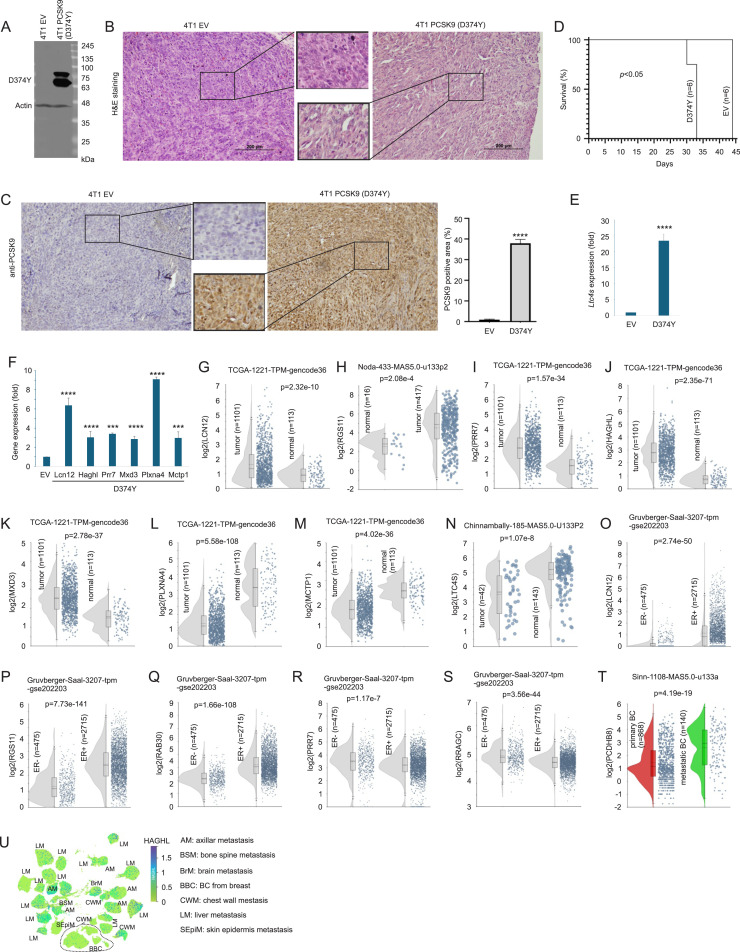
Upregulations of Sig27 component genes in advanced breast cancer. A. Western blot analysis of PCSK9 D374Y and Actin in the indicated stable cell lines: 4T1 EV and 4T1 PCSK9 D374Y. B. H&E staining of 4T1 EV and 4T1 PCSK9 D374Y tumors produced in the mammary fat pad of Balb/c mice (*n* = 6 per group). C. IHC staining and quantification of PCSK9 expression in the indicated tumors. D. Survival curves of mice bearing 4T1 EV or 4T1 PCSK9 D374Y tumors. Statistical analysis was performed using Log-rank (Mantle-Cox) test. E, F. Real-time PCR analysis of the murine counterparts of the indicated Sig27 component genes in EV (4T1 EV) and D374Y tumors. Real-time PCR was performed in triplicates in *n* = 3 per tumor type with 3 repeats and expressed as mean fold change ± SD. Statistical analysis was performed using one-way ANOVA. ***p < 0.001; ****p < 0.0001. G-N. Expression of the indicated Sig27 genes in BC and breast tissues (normal) in the indicated BC cohorts. O-S. Differential expression of the indicated Sig27 genes in ER- and ER+ BCs in the Gruvberger-Saal dataset. T. Upregulation of PCDHB8 in metastatic BC. All analyses were performed using R2: Genomics Analysis and Visualization Platform (http://r2.amc.nl http://r2platform.com). Statistical analyses were carried out using ANOVA provided by the R2 platform. U. HAGHL expression in a single nuclear RNA-seq dataset derived from 60 patients with metastatic BC [21] within the CZ CELLxGENE Discovery website.

The 27-gene panel Sig27 is a predictive biomarker of prostate cancer [14]. To assess its relevance in breast cancer (BC), we examined Sig27’s component genes in PubMed (as of April 19, 2025). Notably, 8 of 27 genes had no prior reports in BC, and another 8 had only limited information (Supplementary Table S4). To investigate the involvement of Sig27’s component genes in BC progression, we utilized our recent finding that the PCSK9 gain-of-function mutant (D374Y) promoted tumorigenesis [15]. Stable 4T1 cell lines expressing D374Y or empty vector (EV) were generated (Fig. 1A) and produced orthotopic tumors in Balb/c mice (Fig. 1B). PCSK9 D374Y overexpression was confirmed (Fig. 1C). D374Y tumors reached endpoint volumes significantly faster than EV tumors (Fig. 1D). The murine counterparts (*Lcn12, Haghl, Prr7, Mxd3, Plxna4, Mctp1, Ltc4s*) of several Sig27 genes with little or no prior BC relevance (Supplementary Table S4) were significantly upregulated in D374Y tumors (Fig. 1E and F).

Further expression analysis in primary BCs revealed upregulation of LCN12, RGS11, PRR7, HAGHL, and MXD3 (Fig. 1G–K), while PLXNA4, MCTP1, LTC4S, PCDHGB2, and PCDHGA5 were downregulated (Fig. 1L–N; Supplementary Fig. S1A–B). The LCN12, RGS11, and RAB30 are expressed at a higher level in ER+ BCs compared to ER- tumors (Fig. 1O–Q), while PRR7 and RRAGC display a reverse pattern (Fig. 1R and S). Interestingly, PLXNA4, LTC4S, and PCDHGB2, despite their reduction in primary BC, were elevated in lymph node metastases (Supplementary Fig. S1C–E), suggesting context- or tumor progression-dependent expression. In this context, PCDHB8 was elevated in metastatic tumors (Fig. 1T). In a comprehensive scRNA-seq (sc: single cell) dataset derived from 60 patients with metastatic BC [21], HAGHL was enriched in malignant cells compared to stromal cells (Supplementary Fig. S1F and G) and in malignant cells across diverse metastatic sites, especially liver (Fig. 1U).

**Fig. 2:**
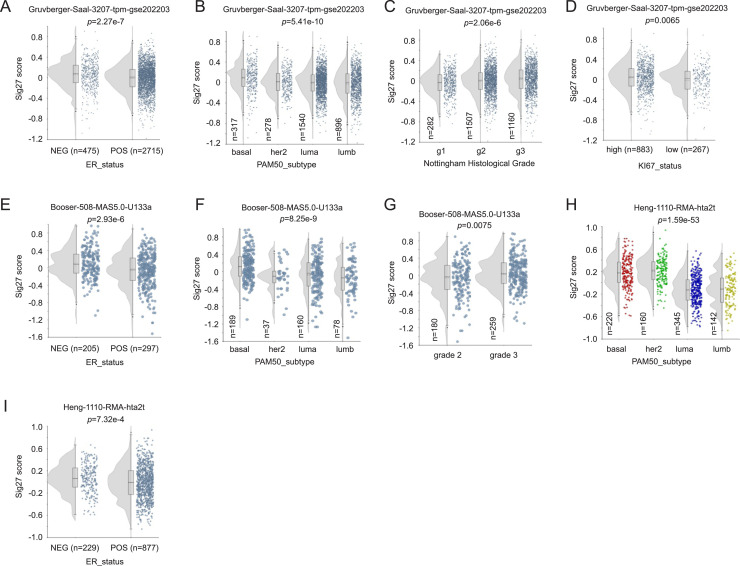
Association of Sig27 metagene with BC. Sig27 metagene expression was presented as Sig27 score calculated by the R2 platform. Elevation of Sig27 score in ER- BCs (A, E, I), basal-like BC (B, F, H), Nottingham Histological Grade BC (C), pathological grade BC (G), and high-proliferative BC (D). Analyses were performed using R2 platform.

Sig27 expression scores were higher in ER− tumors across 9 independent cohorts (*n* = 7408; Fig. 2A, E, I; Supplementary Fig. S2A–F), particularly basal-like subtypes (Fig. 2B, F, H) and tumors with high histological or proliferative grades (Fig. 2C, D, G). Collectively, these data indicate Sig27 as a clinically relevant BC biomarker.

### Sig27 stratifies breast cancer prognosis comparable to oncotypedx and mammaprint

We evaluated Sig27’s prognostic performance in BC, benchmarking it against OncotypeDX and MammaPrint. Gene expression and clinical data were retrieved from three cohorts: METABRIC (*n* = 2509), TCGA (*n* = 1084), and Gruvberger-Sall (GSE202203; *n* = 3207). Risk scores for Sig27, Oncotype DX, and MammaPrint were calculated using cohort-specific Cox regression coefficients: Sigscore = ∑(coef_i_ × Gene_i_ expression) with coef_i_ and Gene_i_ representing gene_i_’s Cox coefficient and gene expression respectively.

**Fig. 3:**
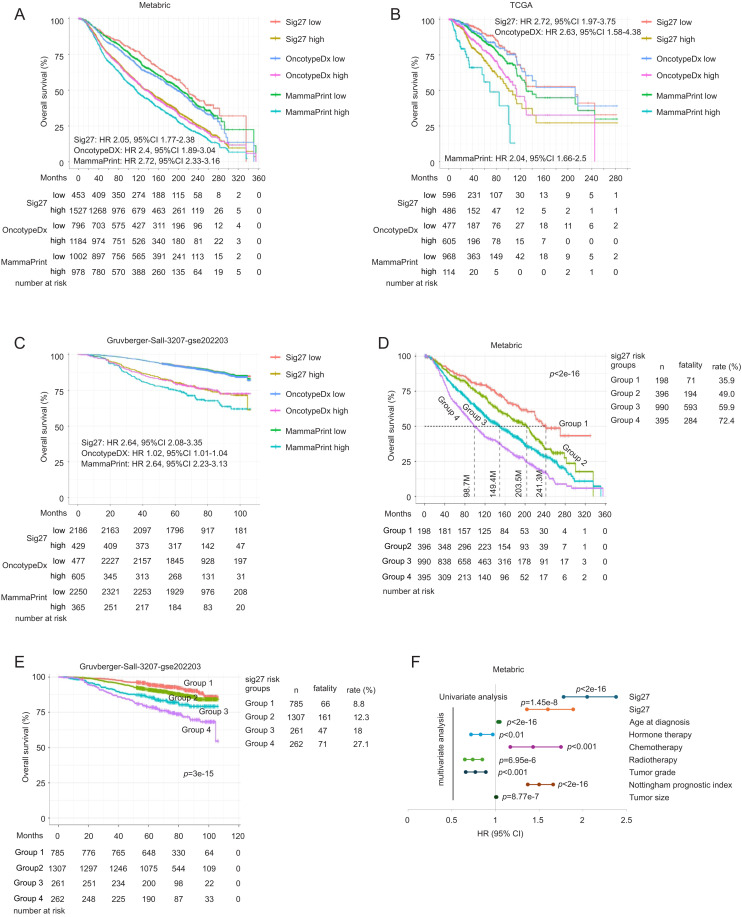
Sig27 predicts BC fatality risk. A-C. Stratification of BC overall survival (OS) probability in three large and independent cohorts using the risk scores of Sig27, Oncotype DX, and MammaPrint. Cutoff points were estimated using Maxstat. The HR and 95 % CI for the risk scores of Sig27, Oncotype DX, and MammaPrint were derived from the indicated datasets. d-E. The indicated datasets were divided into four groups based on Sig27 risk score. The OS probability, patient number, event (death) number, and percentage of deaths in individual groups are presented. F. Univariate and multivariate Cox analyses of Sig27, age at diagnosis (Age), hormone therapy, chemotherapy, radiotherapy, tumor grade, Nottingham prognostic index, and tumor size in assessing OS probability.

Using optimal cutoffs (maxstat), Sig27 stratified BC fatality risk across all cohorts, performing comparably to OncotypeDX (Fig. 3A–C). Sig27 risk score displays comparable hazard ratio (HR) and 95 % CI in evaluating inferior OS compared with OncotypeDX and MammaPrint risk scores (Fig. 3A–C). Additionally, with multiple cutoffs, Sig27 separated patients into low- and high-risk groups with 35.9 % and 72.4 % mortality in the advanced-stage METABRIC dataset (1142/1979 = 57.7 % fatality rate) (Fig. 3D). In the early-stage Gruvberger-Sall cohort (13.2 % fatality), the respective death rates were 8.8 % and 27.1 % (Fig. 3E). Sig27 remained prognostic independent of age, therapy types, tumor grade, size, and the Nottingham Prognostic Index (Fig. 3F).

**Fig. 4:**
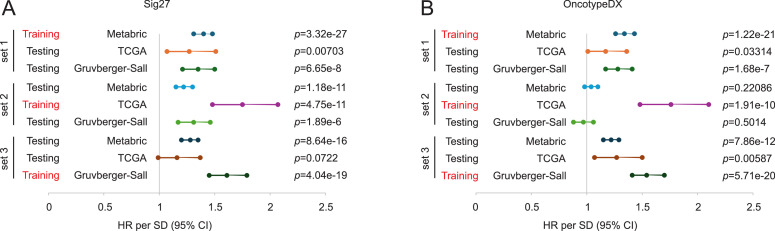
Across-cohort coefficient stability analysis of Sig27. A, B. Across-dataset harmonization was performed using YuGene on the Metabric, TCGA, and Gruvberger-Saal datasets, followed by training and testing in the indicated datasets using fixed coefficients derived from the training dataset. HR per SD (standard deviation) were computed and presented in each training-testing setting for Sig27 (A) and OncotypeDX (B).

We noticed that Sig27 risk scores display similar cohort-specific HRs in assessing OS probability as evidenced by HR 2.05, 95 % CI 1.77–2.38 in METABRIC (Fig. 3A), HR 2.72, 95 % CI 1.97–3.75 in TCGA (Fig. 3B), and HR 2.64, 95 % CI 2.08–3.35 in the Gruvberger-Sall dataset (Fig. 3C), implying that Sig27 captures conserved biological processes with relative association with OS. To determine the coefficient stability of Sig27 risk score across the three cohorts, we performed across-dataset harmonization using YuGene [20], trained Sig27 in each and every dataset, and tested Sig27 in other two datasets (Fig. 4A). The HR derived from the training cohort (METABRIC: HR = 1.40, 95 % CI 1.31–1.48) was preserved in two independent validation cohorts (TCGA: HR = 1.27, 95 % CI 1.07–1.51; GSE202203: HR = 1.35, 95 % CI 1.21–1.50) (Fig. 4A). Similar observations were obtained in other two training-testing settings (Fig. 4A). The comparable effect sizes, overlapping CIs, and consistent directionality support the stability and transferability of Sig27 score’s Cox coefficient and argue against substantial overfitting. HRs attenuate modestly from training to external validation, as expected, but consistently retain magnitude, directionality, and overlapping CI (Fig. 4A). We also performed the sensitivity analysis on OncotypeDX (Fig. 4B). Sig27 exhibits better cross-cohort stability than OncotypeDX (Fig. 4A and B). Collectively, across three independent training–testing permutations using harmonized expression data, Sig27 displays stable and transferable score-level Cox coefficients. These observations support biological robustness of the underlying process captured by Sig27 and oppose material overfitting, while acknowledging moderate context-dependent variation in effect size. These analyses provide indirect validation of Sig27, despite its prostate cancer origin [14], as an effective biomarker in BC.

**Fig. 5:**
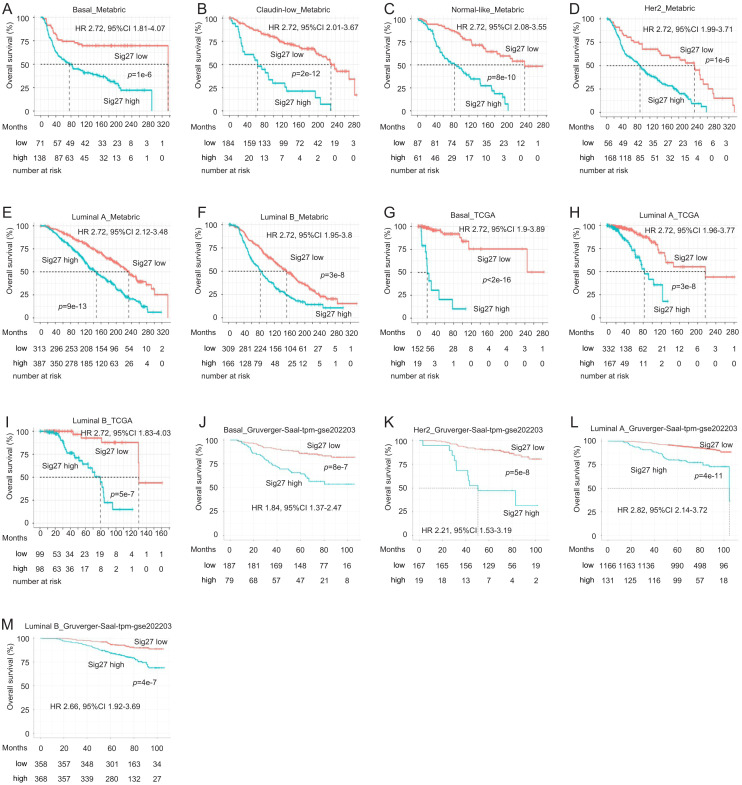
Sig27 stratifies the fatality risk among intrinsic subtypes of BC. The Metabric, TCGA, and Gruvberger-Saal datasets were utilized. Cutoff points were estimated using Maxstat. HR and 95 % CI for Sig27 risk scores in the indicated intrinsic BC subtypes within the indicated datasets are shown.

Given that the 4T1 orthotopic allografts are a model of TNBC and the mixture of BC subtypes in the above BC datasets, we analyzed the predictive biomarker potential of Sig27 in individual intrinsic BC subtypes. Sig27 stratified poor OS in basal-like BC in the METABRIC (Fig. 5A), TCGA (Fig. 5G) and Gruvberger-Sall cohorts (Fig. 5J), as well as in other intrinsic BC subtypes (Claudin-low, HER2-enriched, Luminal A/B, and Normal-like) in each dataset (Fig. 5B–F, H–I, and K-M). This indicates that Sig27 reflects the same or similar biological signals which influence poor OS across intrinsic BC subtypes. Nonetheless, Sig27 appears to strongly predict inferior OS in basal-like BC compared to other intrinsic subtypes, consistent with Sig27’s association with 4T1 D374Y orthotopic allografts (Fig. 1A-F).

### Sig27 captures BC’s immunosuppressive features

**Fig. 6:**
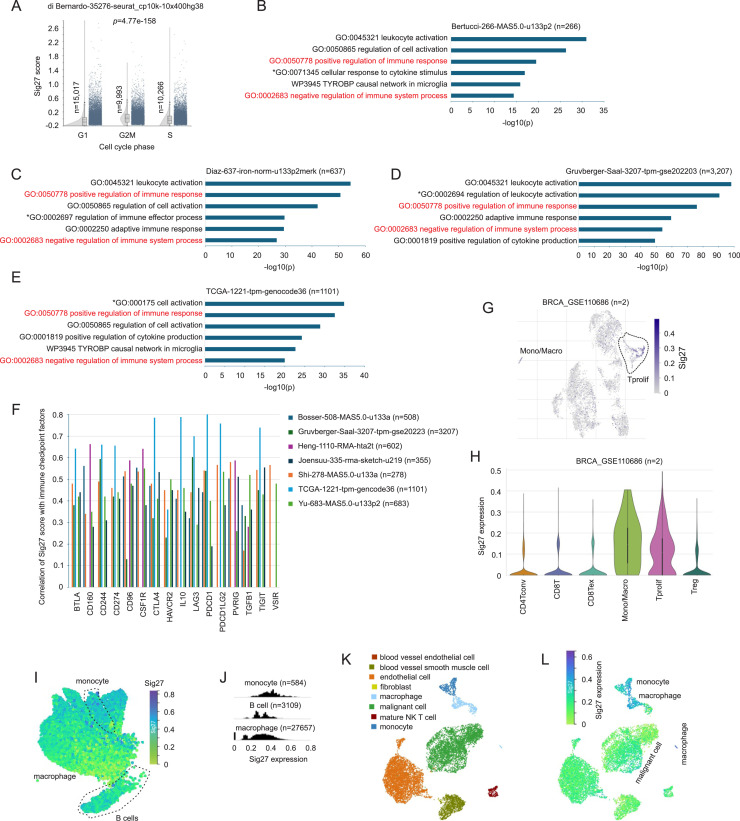
Sig27 is associated with tumor immunity. A. Sig27 metagene expression was analyzed in G1, G2/M, and S phase in 35,276 cells across 32 BC cell lines in the indicated sc-RNA-seq dataset within the R2 platform. B-E. Genes correlated with Sig27 metagene were obtained from the indicated datasets with R2. Top positively correlated genes (*r* ≥ 0.45, *n* < 600, and *p* < 0.0001) were analyzed for pathway enrichment using Metascape [18]. The top 6 enrichments are graphed. Pathways marked in red and * are conserved and unique respectively in the four datasets. The rest of pathways exist in more than one dataset. F. Correlations of Sig27 with the indicated ICs in multiple BC cohorts. Only those ICs correlated with Sig27 at *r* ≥ 0.5 in at least one dataset are graphed. G-H. Sig27 metagene expression, and quantification are shown in the BRCA_GSE11086 scRNA-seq dataset (see Supplementary Fig. S4A for cell map). Analyses were performed using the TISCH2 platform [17]. I-J. Sig27 metagene expression in monocytes, macrophages, and B cells in metastatic BCs. K-L. Cell map and Sig27 expression in liver metastasis of BC. The analysis was performed using a single nuclear RNA-seq dataset derived from 60 patients with metastatic BC [21].

We investigated BC’s biological axis captured by Sig27. In the di Bernardo scRNA-seq dataset (35,276 cells from 32 BC cell lines), the OncotypeDX and MammaPrint metagenes were highly enriched in G2/M-phase cells, reflecting their association with proliferation (Supplementary Fig. S3A and B). Sig27 expression, in contrast, showed a lesser enrichment in G2/M-phase cells (Fig. 6A).

To determine BC’s biological features associated with each panel, we identified genes significantly correlated with Sig27, Oncotype DX, and MammaPrint metagenes across four independent cohorts (*n* = 5211 tumors). Genes positively correlated with OncotypeDX and MammaPrint were consistently enriched in mitotic and cell cycle pathways (Supplementary Fig. S3C–J). By contrast, Sig27-correlated genes were commonly enriched in immune-related pathways across all cohorts, exhibiting overlapping and cohort-specific enrichments (Fig. 6B–E).

Notably, Sig27 demonstrated strong correlations with immune checkpoint molecules—including PD-1, CTLA4, IL-10, LAG3, and TIGIT, across 7 datasets covering 6734 tumors (Fig. 6F). Single-cell analysis revealed Sig27 expression predominantly in tumor-associated macrophages (TAM), with additional presence in regulatory T cells (Tregs) and exhausted CD8+ *T* cells (CD8Tex) (Fig. 6G–H), confirmed by expression of CSF1R, CTLA4, PD-L1, HAVCR2, and TIGIT (Supplementary Fig. S4A–F). In 60 metastatic BCs, Sig27 was highly expressed in macrophages, monocytes, and B cells (Fig. 6I–J; Supplementary Fig. S5A). In typical liver metastasis, Sig27 expression was highly enriched in macrophages and monocytes, while its expression was in a lower level in malignant cells (Fig. 6K–L) [21]. Similar expression pattern was also detected in the other 6 liver metastases (data not shown) [21].

These observations suggest Sig27 is associated with the immunosuppressive features of BC, distinct from mainly proliferation-based signatures like OncotypeDX and MammaPrint.

### Sig27 predicts BC’s response to immune checkpoint inhibitor therapy

**Fig. 7:**
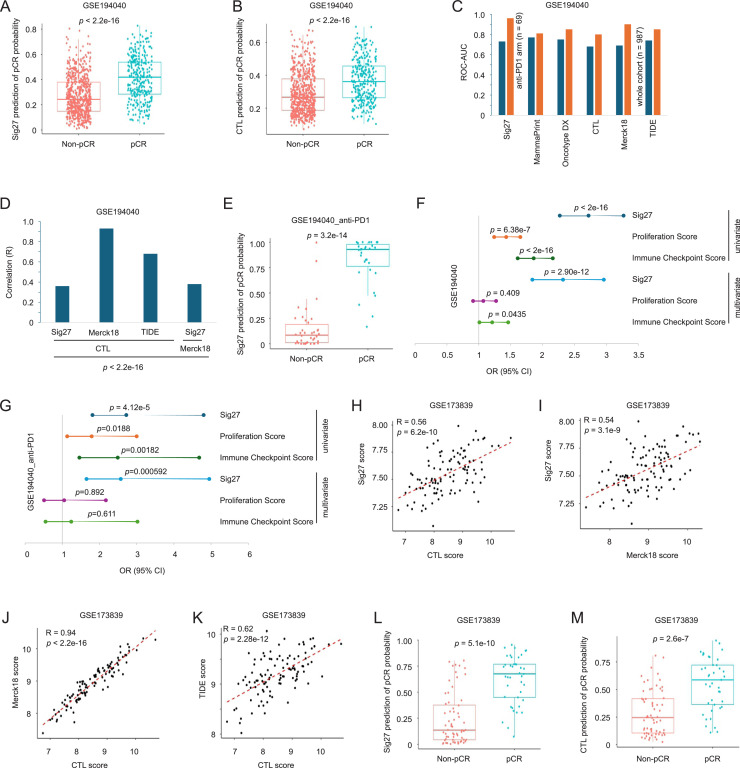
Sig27 predicts pCR in BC treated with immune checkpoint inhibitor (ICI) therapy. A, B. Logistic regression modeling of Sig27 and CTL in predicting the probability of pCR in the GSE194040 dataset. C. ROC-AUC evaluation of Sig27 and the indicated benchmark signatures in predicting pCR using logistic regression in the intact GSE194040 dataset and the sub-dataset of PD1 inhibitor treatment. D. Pearson correlations of Sig27, Merck18, and TIDE with CTL as well as correlation of Sig27 with Merck18. E. Logistic regression modeling of Sig27 in predicting pCR in the PD1 inhibitor subpopulation of GSE194040. F, G. Univariate and multivariate logistic regression of Sig27, Proliferation Score and Immune Checkpoint Score toward pCR in the indicated datasets. H-K. Pearson correlations between the indicated gene sets in the GSE173839 cohort. L, M. Logistic regression modeling of Sig27 and CTL in predicting the probability of pCR in the GSE173839 dataset.

By using gene expression data from the I-SPY2 trial, an ongoing phase II neoadjuvant clinical trial (NCT01042379, https://clinicaltrials.gov/study/NCT01042379) on high-risk and early-stage BCs for pathologic complete response (pCR) treated with different novel drugs [22], we downloaded GSE194040 (*n* = 987), which consisted of 10 treatment arms, including a PD1 inhibitor (anti-PD1 pembrolizumab) across the three major histological BC subtypes [23]. The Sig27-derived prediction of pCR was then analyzed and benchmarked against MammaPrint, OncotypeDX, as well as three established tumor inflammation gene sets: cytotoxic T lymphocyte (CTL) [24], Merck18 (tumor inflammation signature/TIS) [25], and TIDE [24]. Logistic regression revealed that Sig27, CTL, MammaPrint, OncotypeDX, Merck18, and TIDE predict pCR (Fig. 7A and B; Supplementary Fig. S6A–D). Sig27 predicts pCR comparably or superior to MammaPrint, OncotypeDX, CTL, Merck18, and TIDE (Fig. 7C). Compared to MammaPrint and OncotypeDX (Supplementary Fig. S6E and F), Sig27 shows a stronger correlation with CTL (Fig. 7D) and displays significant correlation with Merck18 (Fig. 7D). As expected, Merck18 and TIDE are highly correlated with CTL (Fig. 7D). These analyses thus suggest that Sig27 contains an immune component relevant to tumor inflammation. The complex neoadjuvant treatments in GSE194040 imply that prediction of pCR using tumor inflammation gene sets (CTL, Merck18, and TIDE) and proliferation signatures (MammaPrint and Oncotype DX) operate via different tumor properties. Given no meaningful correlations of MammaPrint and OncotypeDX with CTL (Supplementary Fig. S6E and F), their prediction of pCR might be primarily attributed to the tumor’s proliferative feature. Sig27, on the other hand, likely predicts pCR partially via tumor’s immune properties.

To investigate this concept, we observed that Sig27 increased its prediction of pCR in BC treated with pembrolizumab (Fig. 7E). Its performance in BCs treated with anti-PD1 is either comparable or superior to other benchmark signatures, as revealed by ROC-AUC (Receiver Operating Characteristic-Area Under the Curve) analysis (Fig. 7C).

Given Sig27’s association with BC’s immunosuppression properties (Fig. 6B–L) and cell proliferation (Fig. 6A), we analyzed Sig27-derived prediction of pCR in the context of immune score and proliferation score. Univariate logistic regression revealed that Sig27, Proliferation Score (ProScore), and Immune Checkpoint Score (ICS) all positively associated with pCR (Fig. 7F and G). When analyzed by multivariate logistic regression, Sig27 remained strong associations with pCR with a slightly attenuated Odds Ratio (OR) along with loss of significance for ProScore and ICS in either or both GSE194040 and GSE194040_anti-PD1 (Fig. 7F and G), indicating that Sig27 captures the associations of both ICS and ProScore with pCR.

We further analyzed GSE173839 (*n* = 372) also from the I-SPY2 trial, which consisted of TN and HR+/HER2- BCs treated with the neoadjuvant regimens of anti-PD-L1 (durvalumab) with PARP inhibitor Olaparib plus Taxol and the standard-of-care Ctrl T-AC (Taxol followed by doxorubicin and cyclophosphamide) chemotherapy [26]. Both MammaPrint and OncotypeDX show either no or marginal negative correlation with CTL (Supplementary Fig. S6G and H). In comparison, Sig27 displays a high-level association with CTL (Fig. 7H and I). Merck18 and TIDE are highly correlated with CTL (Fig. 7J and K). Sig27, CTL, MammaPrint, Oncotype DX, Merck18, and TIDE predict pCR (Fig. 7L and M; Supplementary Fig. S6I–L). Collectively, the above evidence suggests Sig27’s immune component likely contributes to its biomarker potential.

### Sig27IM: a refined immunosuppressive gene panel in breast cancer

**Fig. 8:**
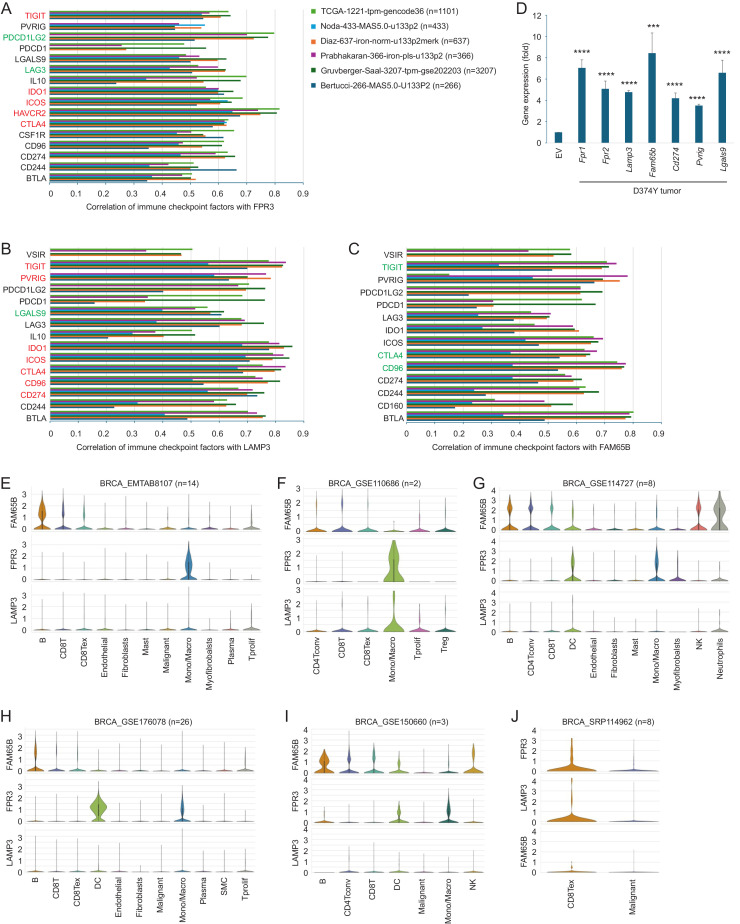
Robust association of FPR3, LAMP3, and FAM65B with the immunosuppressive features of BC. A-C. Correlations of FPR3, LAMP3, and FAM65B with the indicated ICs in the indicated BC datasets. Only those ICs correlated with FPR3, LAMP3, or FAM65B at *r* ≥ 0.5 in at least one dataset are listed. ICs marked red show correlation at *r* ≥ 0.5 in all 6 datasets; ICs marked green display correlations at *r* ≥ 0.5 in 5 out of 6 datasets. Analyses were performed using the R2 platform. D. Upregulations of Sig27 component genes (murine counterparts *Fpr1, Fpr2, Lamp3,* and *Fam65b*) and murine IC genes: *Cd274, Pvrig,* and *Lgals9* in 4T1 PCSK9 D374Y (D374Y) tumors compared to 4T1 EV tumors. E-J. Expression of FAM65B, FPR3, and LAMP3 in the indicated cell population within the indicated scRNA-seq datasets. Analyses were performed using the TISCH2 platform.

We further polished the immune component of Sig27. Among Sig27 genes, we identified FPR3, NOD2, and LAMP3, which are known for roles in immune recognition and antigen presentation, implying Sig27’s immune relevance. Specifically, FPR3 and NOD2 are pattern recognition receptors (PRRs) [27,28]. LAMP3 plays a role in antigen presentation in dendritic cells [29]. Screening Sig27 components in 6 cohorts (*n* = 6010) revealed FPR3, LAMP3, and FAM65B strongly associated with immune checkpoints (ICs) such as PD-L1, CTLA4, PVRIG, and LGALS9 (Fig. 8A–C). While ICs (like PD-L1 and LGALS9) can be expressed in tumor cells, ICs are commonly expressed in immune cells and contribute to cancer immune evasion. As expected, all above 6 BC cohorts were infiltrated with a complex set of immune cell populations (Supplementary Table S5A–F). The commonly infiltrated CD8+ *T* and Treg cells across datasets (Supplementary Table S5A–F) support the involvement of PD-1 (PDCD1), CTLA4, HAVCR2, and TIGIT in CD8+ *T*-cell exhaustion and Treg’s immune suppressive action. M2 macrophages were the major immune cells present in BCs across all 6 BC datasets (Supplementary Table S5A–F) and are the dominant immunosuppressive macrophage subset which contributes to CD8+ *T*-cells exhaustion and cancer immune evasion [30]. CSF1R (colony-stimulating factor 1 receptor) promotes tumor-associated macrophages (TAM) survival, proliferation, and M2 polarization and contributes to cancer immune evasion [31]. The observed high-level correlations of FRP3, LAMP3, and FAM65B with the set of immune checkpoints in the 6 BC datasets (Fig. 8A–C) implies the association of FRP3, LAMP3, and FAM65B with BC immune evasion. These associations were mirrored in advanced 4T1 D374Y mouse tumors, which showed upregulation of murine homologs (*Fpr1/2, Lamp3, Fam65b*) and IC genes (*Cd274, Pvrig, Lgals9*) (Fig. 8D). The mouse gene of human *FPR3* remains unclear [27]; evidence nonetheless indicates that both mouse *Fpr1* and *Fpr2* genes perform similar functions [27,32]. Binding of LGALS9 (encoding Galactin-9) to its receptor HAVCR2 (TIM-3) contributes to immunological tolerance [33]. FAM65B, though unstudied in BC, correlated with ICs that inhibit CD226 signaling (TIGIT, PVRIG, CD96) [[34], [35], [36]], suggesting its role in immune suppression. Single-cell RNA-seq data showed FAM65B expression in B cells, CD8Tex, and Tregs (Fig. 8E–J). In metastatic BCs, FAM65B (RIPOR2) is prominently expressed in B cells, T cells, and monocytes (Supplementary Fig. S5C). FPR3 and LAMP3 localized mainly to TAM and CD8Tex (Figs. 8E–J and S5B). The status of TAM, CD8Tex, and Treg cells was confirmed by the expression of CSF1R, CTLA4, PD-1 (PDCD1), HAVCR2, and TGIT (Supplementary Fig. S7A–F).

**Fig. 9:**
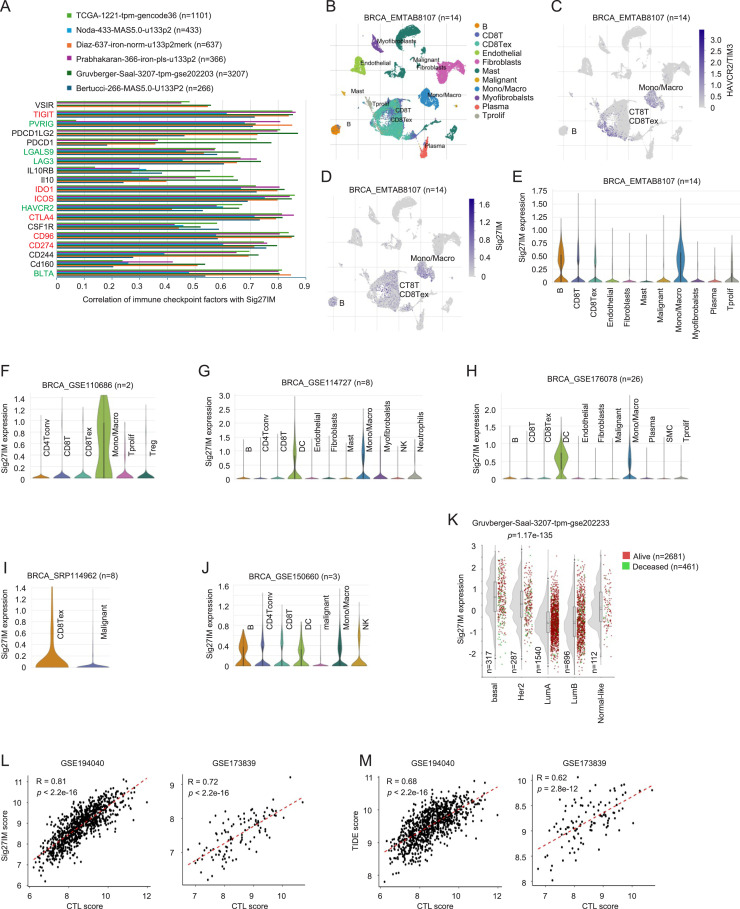
Sig27IM displays strong associations with immunosuppressive features of BC. A. Correlations of Sig27IM with the indicated ICs in the indicated BC datasets. Only those ICs correlated with Sig27IM at *r* ≥ 0.5 in at least one dataset are listed. ICs marked red show correlation at *r* ≥ 0.5 in all 6 datasets; ICs marked green display correlation at *r* ≥ 0.5 in 5 out of 6 datasets. Analyses were performed using the R2 platform. B-J. Sig27IM expression in the indicated cell population within the indicated scRNA-seq datasets. Analyses were performed using the TISCH2 platform. K. Elevations of Sig27IM metagene expression in basal-like BC. Analysis was performed using the R2 platform. L, M. Correlations of Sig27IM (L) and TIDE (M) with CTL in the indicated datasets.

We grouped *FPR3, LAMP3*, and *FAM65B* genes into an immune-focused panel, Sig27IM, which displayed stronger correlations with ICs (Fig. 9A) than Sig27 across multiple datasets (Fig. 6F). In scRNA-seq data, Sig27IM is enriched in TAM, CD8Tex, dendritic, and B cells (Figs. 9B–J and S5D). Sig27IM expression is elevated in basal-like and HER2-enriched subtypes (Figs. 9 K and S8), both associated with poor prognosis. Furthermore, in both the GSE194040 and GSE173839 cohorts of the I-SPY2 trials involving PD1 and PD-L1 neoadjuvant regimens, respectively [23,26], Sig27IM exhibits robust correlations with CTL (Fig. 9L). These correlations exceed those observed for TIDE, a well-established biomarker of tumor inflammation [24] in relation to CTL (Fig. 9M). The high-level association of Sig27IM with tumor inflammation features as evidenced by CTL is validated by its impressive correlation with Merck18 (Supplementary Fig. S9A). Compared to Sig27 (Fig. 7D and F), Sig27IM correlates with CTL at higher levels (Fig. 9L). While Sig27IM predicts responders (pCR) in both GSE194040 and GSE173839 (Supplementary Fig. S9B), Sig27 is superior in discriminating responders (Supplementary Fig. S9C and D), indicating that Sig27 stratifies pCR via additional biological axis besides its Sig27IM-derived immune component. The existence of immune components in Sig27 and its enrichment in Sig27IM were additionally validated by the substantial increases in correlation with the STAT1_sig (Supplementary Fig. S9E–H). STAT1_sig was shown to be strongly associated with pCR in BCs treated with PD1 and PD-L1 inhibitors in GSE194040 [23] and GSE173839 [26]. Together, these findings suggest Sig27IM as an enhanced model of BC immunosuppression compared to Sig27.

### Validation of Sig27 as a pan-cancer prognostic biomarker

**Fig. 10:**
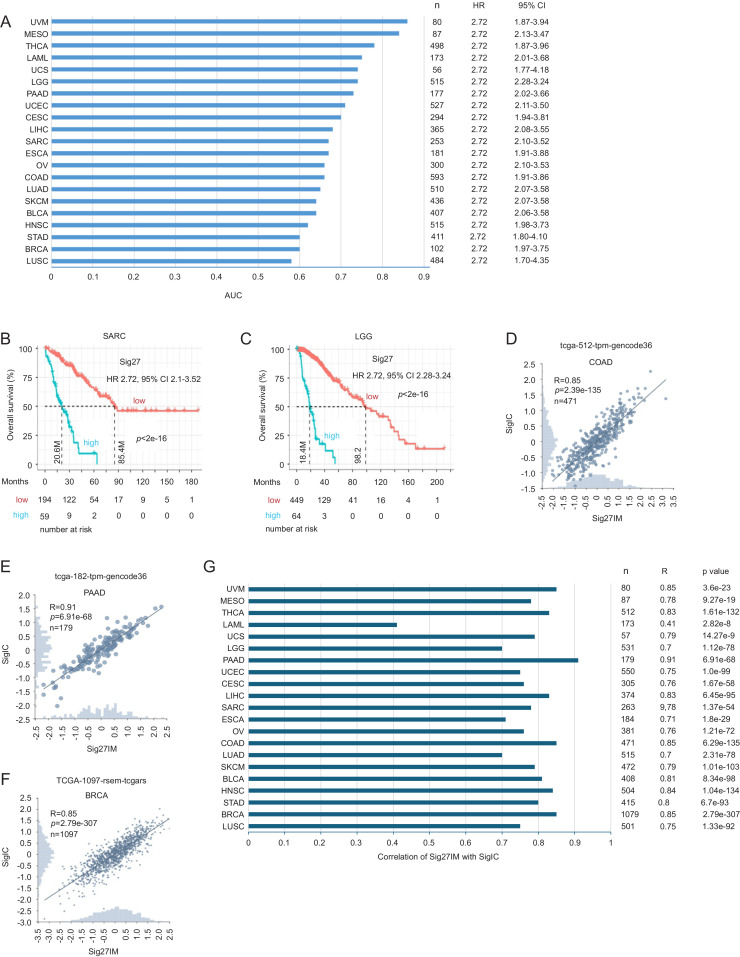
Sig27 effectively assesses poor prognosis in pan-cancer. A. The AUC values of Sig27 in discriminating prognostic outcome in the indicated cancer types. The size (n) of TCGA PanCancer datasets used in these analyses is indicated. The TCGA datasets used for these analyses include - BLCA: bladder urothelial carcinoma; BRCA: breast invasive carcinoma; CESC: Cervical squamous cell carcinoma; COAD: colon adenocarcinoma; ESCA: esophageal carcinoma; HNSC: head and neck squamous cell carcinoma; LAML: acute myeloid leukemia; LGG: brain lower grade glioma; LIHC: liver hepatocellular carcinoma; LUAD: lung adenocarcinoma; LUSC: lung squamous cell carcinoma; MESO: mesothelioma; OV: ovarian serous cystadenocarcinoma; PAAD: pancreatic adenocarcinoma; SARC: sarcoma; SKCM: skin cutaneous melanoma; STAD: stomach adenocarcinoma; THCA: thyroid carcinoma; UCEC: uterine corpus endometrial carcinoma; UCS: uterine carcinosarcoma; and UVM: uveal melanoma. B, C. Sig27-mediated stratifications of OS probability in SARC and LGG. d-G. Correlations of Sig27IM with SigIC in the indicated cancer types. Analyses were performed using the respective TCGA dataset within the R2 platform.

Immune evasion is a hallmark of cancer. Given the association of Sig27 with immunosuppressive properties in BC, we demonstrated that the cohort-specific Sig27 risk scores exhibited comparable HR (Fig. 10A), suggesting relatively similar influence of tumor immunosuppressive properties, likely captured by Sig27, on tumor aggressiveness across these cancer types. Nonetheless, Sig27 stratifies poor OS risk with an AUC > 0.5 across 21 cancer types (*n* = 7808), including breast cancer (BC) (Fig. 10A). The AUC variations from around 0.6 to above 0.8 reflect tumor-specific immune architecture, baseline of immune infiltration, the strength of association between tumor immunity and tumor aggressiveness, heterogenous biological axis, treatment variations, and unique follow-up systems of individual cancer types. Sig27 risk score-derived separation of sarcoma (SARC) and lower-grade glioma (LGG) into low- and high-risk groups is evident (Fig. 10B and C). Collectively, these findings suggest Sig27 as a novel and effective biomarker for predicting poor prognosis in BC and other cancers.

Given the likelihood that Sig27IM, a three gene subset of Sig27, contributes to the immunosuppressive properties of Sig27, we demonstrated robust correlations between Sig27IM and SigIC across 21 cancer types (except LAML) for which poor prognosis was significantly predicted by Sig27 (Fig. 10A–C), including BRCA (breast cancer), COAD (colon adenocarcinoma), and PAAD (pancreatic adenocarcinoma) (Fig. 10D–G). SigIC consists of 22 established immune checkpoints and strongly associates with cancer immune evasion [37]. Collectively, these observations suggest the general association of Sig27IM with cancer’s immunosuppressive properties and provide an additional support for Sig27 predicting poor OS in BC and other cancer types partially via capturing the relevant immune evasion property of cancer.

## Discussion

Women’s health lies at the heart of society, and breast cancer remains the leading health threat. Although BC is most extensively studied, with significant advances in risk assessment and treatment, clinical management, particularly in risk prediction, remains in need of improvement. Our study addresses this gap through a cross-cancer biomarker approach, utilizing Sig27, a signature originally derived from prostate cancer, to assess risk stratification in BC.

While cancer heterogeneity supports tumor-type-specific biomarkers, shared hallmarks such as proliferation and immune evasion [38] justify pan-cancer investigations. Sig27 harbors immune-relevant components, notably FPR3 and NOD2, both pattern recognition receptors [27,28], and has previously modeled immunosuppressive features in renal cell carcinoma [39]. We hypothesized that Sig27 could assess BC fatality risk partially via its immune signaling relevance, offering a more objective biomarker approach than traditional, cancer-type-restricted signatures.

Our findings validate this strategy: Sig27 performs comparably to the established multigene panels OncotypeDX and MammaPrint, though it captures distinct tumor properties. Unlike OncotypeDX and MammaPrint, which are mainly proliferation-focused [11,12], Sig27 is enriched in immune-related pathways and strongly correlates with immune checkpoint (IC) genes and immunosuppressive cells in both primary and metastatic BCs.

Further, Sig27IM, a three-gene immune module within Sig27 (*FPR3, LAMP3*, and *FAM65B*), robustly associates with key ICs including PD-L1 (CD274), LGALS9, PVRIG, and CTLA4. Notably, orthotopic 4T1 BC tumors expressing mutant PCSK9 (D374Y) showed upregulation of Sig27IM genes and multiple ICs, providing in vivo validation of the signature’s immunological significance. Nonetheless, the 4T1 orthotopic allografts are a murine model of TNBC. Given the different properties of individual BC subtypes, Sig27’s immunosuppressive features supported by the 4T1 D374Y tumors may not equivalently capture the counterpart features in other BC subtypes.

FAM65B (RIPOR2), largely uncharacterized in BC, emerged as a potential contributor to immune evasion, showing strong IC associations and specific expression in immune cell subsets. Our results indicate FAM65B (RIPOR2) as a potential candidate for future BC immunotherapy studies as well as are consistent with FAM65B being expressed in T cells and suppressing T cell activation [40].

While our findings collectively suggest Sig27, particularly Sig27IM, as promising immune-based biomarkers for BC, the retrospective nature of our study is a limitation with respect to bias in patient selection and unaccounted confounding variables. In this regard, the findings reported here require prospective validations. Given the association of Sig27 with 4T1 D374Y tumors and its strong performance in stratifying inferior OS in basal-like BCs across the METABRIC, TCGA, and Gruvberger-Sall datasets, it is tempting to suggest that validations could focus on TNBC on both neoadjuvant and adjuvant platforms. The relevance of this translational roadmap includes 1) basal-like BC consists of approximately 80 % of TNBC [41], 2) TNBC is the most immunogenic BC subtype with higher levels of tumor-infiltrating immune cells, elevated tumor mutation burden and PD-L1 expression [42,43], 3) the PD-1 inhibitor Pembrolizumab is involved in neoadjuvant and adjuvant treatments [44], and 4) PD-L1 expression also predicts response to the commonly used neoadjuvant chemotherapy [45]. Additionally, the relationship between Sig27 and the established immune scores (immunescore [46]) in BC should be integrated into these validation efforts.

## Ethics approval

Our research utilized retrospective datasets from TCGA and other publicly available datasets (see Supplementary Table S4). Animal experiments were performed following the protocols approved by the McMaster University Animal Research Ethics Board (AUP#: 24-04).

## Funding

This research was supported by a 10.13039/501100007202CIHR grant (202309PJT-506896-C2-CEBA-108276) to DT as well as by grants from Heilongjang Provincial Foreign Experts for the year 2024 (G2024044) to TZ and The Natural Science Foundation of Heilongjiang Province of China (PL2024H020) to TZ. All funding sources were not involved in research design, data collection, interpretation, and analysis; in manuscript preparation; as well as in finalizing the decision to submit this manuscript for publication.

## Declaration of AI-assisted technologies in the writing process

During manuscript revision, we used ChatGPT and Copilot to improve the English presentation. After using these tools, we reviewed and edited the content and take full responsibility for the content of this manuscript.

## CRediT authorship contribution statement

**Yingying Su:** Writing – review & editing, Writing – original draft, Investigation, Formal analysis, Data curation, Conceptualization. **Ying Dong:** Writing – review & editing, Formal analysis. **Tao Zhang:** Writing – review & editing, Formal analysis, Conceptualization. **Damu Tang:** Writing – review & editing, Writing – original draft, Supervision, Funding acquisition, Formal analysis, Data curation, Conceptualization.

## Declaration of competing interest

All authors declare no competing interests.

## Data Availability

All data utilized and produced in this study are included in Supplementary materials and are available upon request.

## Appendix Supplementary materials

Image, application 1

Image, application 2

Image, application 3
