# Supplementary material for: The Sig27 multigene stratifies breast cancer fatality risk via reflecting tumor-associated immune suppressive features
Source: Transl Oncol. 2026 May 18;69:102820. doi: 10.1016/j.tranon.2026.102820 (PMC13202559; doi:10.1016/j.tranon.2026.102820)
Supplement: Supplementary file 1 [file mmc1.pdf]

Supplementary Figure S1

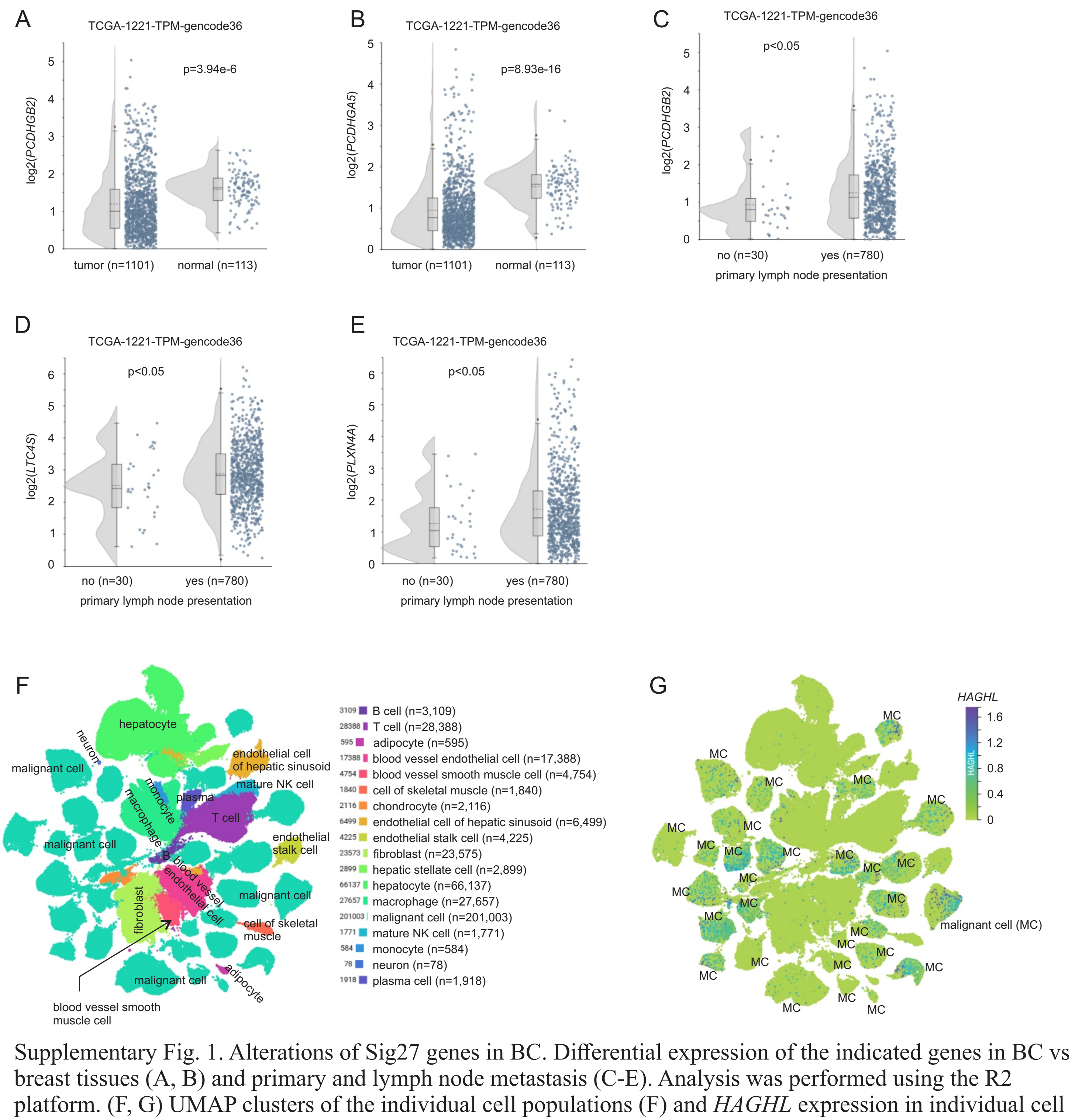

Supplementary Fig. 1. Alterations of Sig27 genes in BC. Differential expression of the indicated genes in BC vs breast tissues (A, B) and primary and lymph node metastasis (C-E). Analysis was performed using the R2 platform. (F, G) UMAP clusters of the individual cell populations (F) and *HAGHL* expression in individual cell populations. Analysis was performed using "HTAN/HTAPP Broad - Spatio-molecular dissection of the breast cancer metastatic microenvironment" within CELLxGENE Discovery website (accessed on July 14, 2025). The dataset was composed of 60 patients with BC metastasized to brain, neck, chest wall, axilla, lung, liver, skin, and bone. The single nuclear RNA-seq dataset with BCs from axilla, bone, brain, breast, chest wall, liver, and skin epidermis was utilized.

Supplementary Figure S2

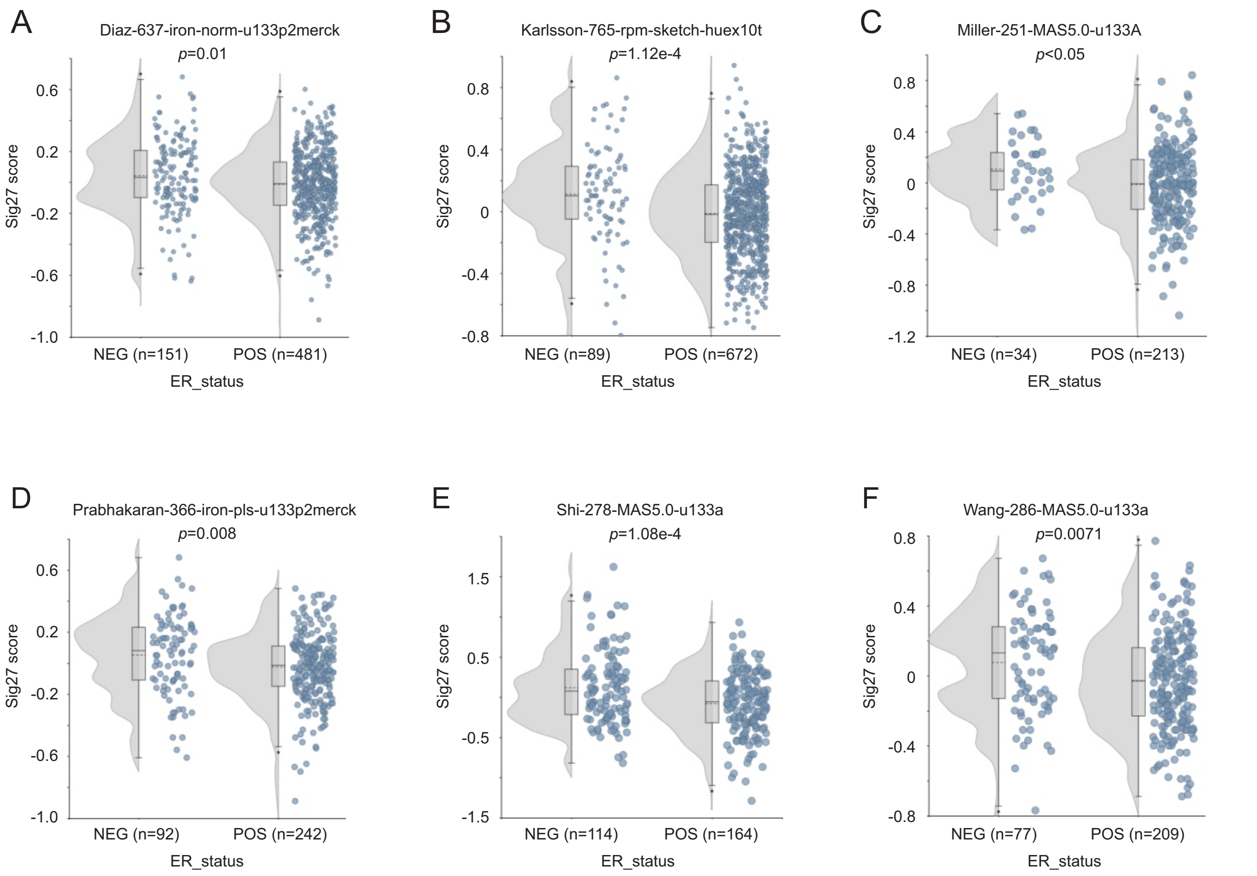

Supplementary Fig. 2. Differential expression of Sig27 in BC. Sig27 metagene expression was measured using Sig27 score calculated using z score by the R2 platform. Elevations of Sig27 metagene expression in ER- BCs are shown in the indicated datasets. Analysis was performed using the R2 platform.

Supplementary Figure S3

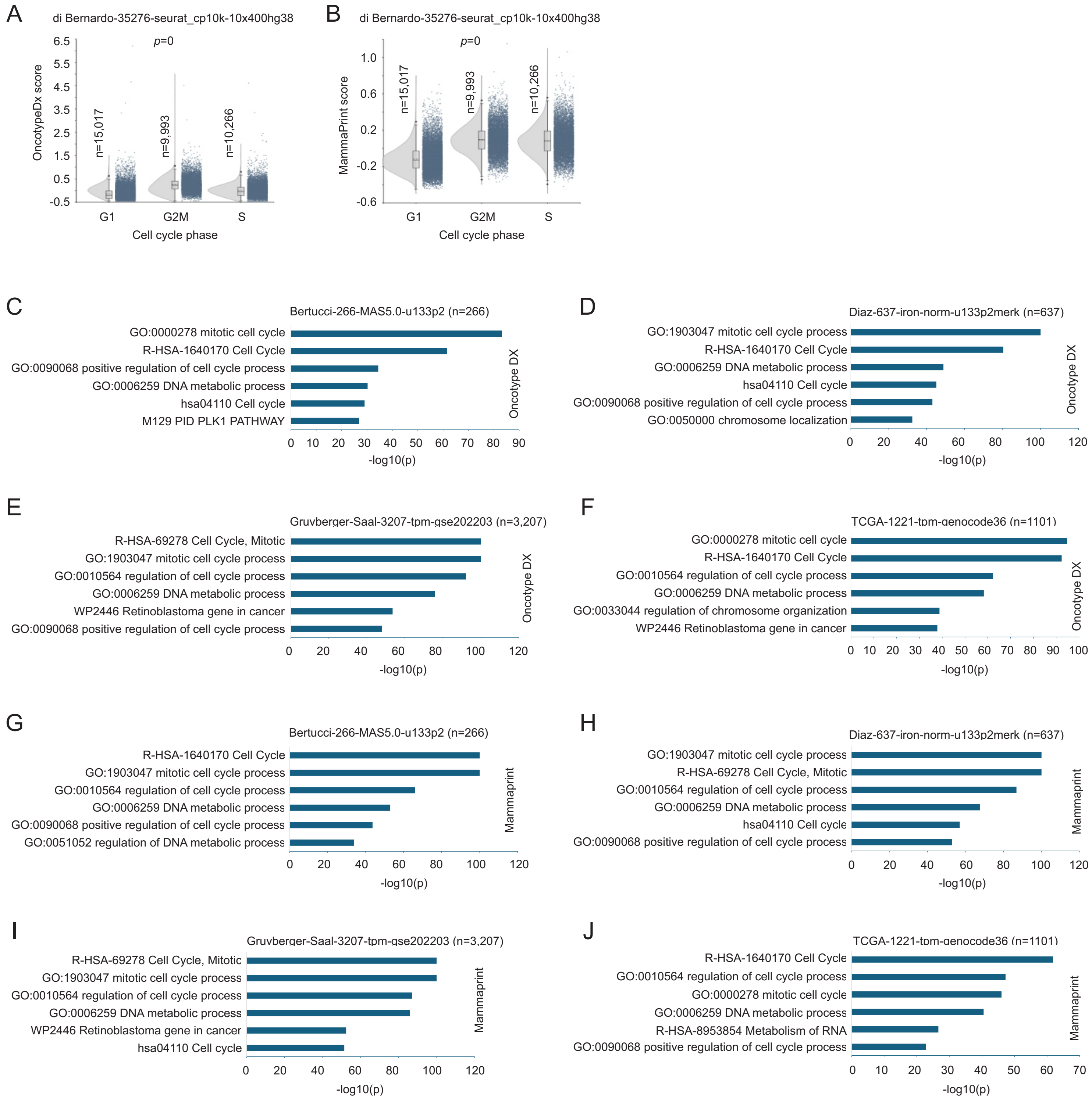

Supplementary Fig. 3. Association of Oncotype DX and Mammaprint with mitotic cell cycle progression. A-B. Oncotype DX and Mammaprint metagene expressions (scores) was present at higher levels in G2/M, which was determined in 35,276 cells across 32 BC cell lines. Analysis was performed using the R2 platform. C-J. Genes correlated with the Oncotype DX and Mammaprint metagenes were obtained from the indicated datasets within R2. Top positively correlated genes ($r \geq 0.45$, $n<600$, and $p<0.0001$) were analyzed for pathway enrichment using Metascape for Oncotype DX (C-F) and Mammaprint (G-J).

Supplementary Figure S4

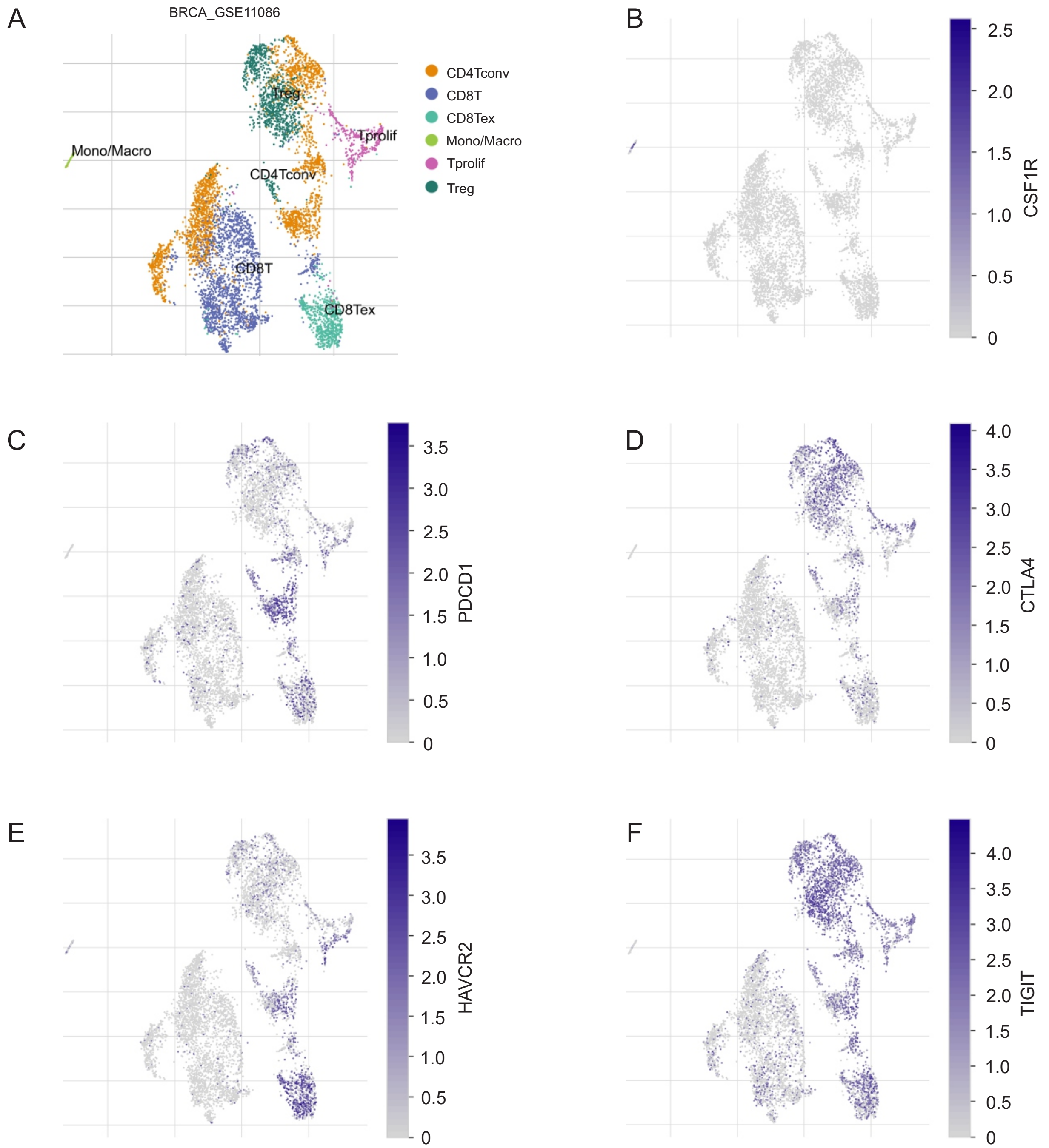

Supplementary Fig. 4. Expression of the indicated ICs in single cell populations in the BRCA_GSE11086 scRNA-seq datasets. Analyses were performed using the TISCH2 platform.

Supplementary Figure S5

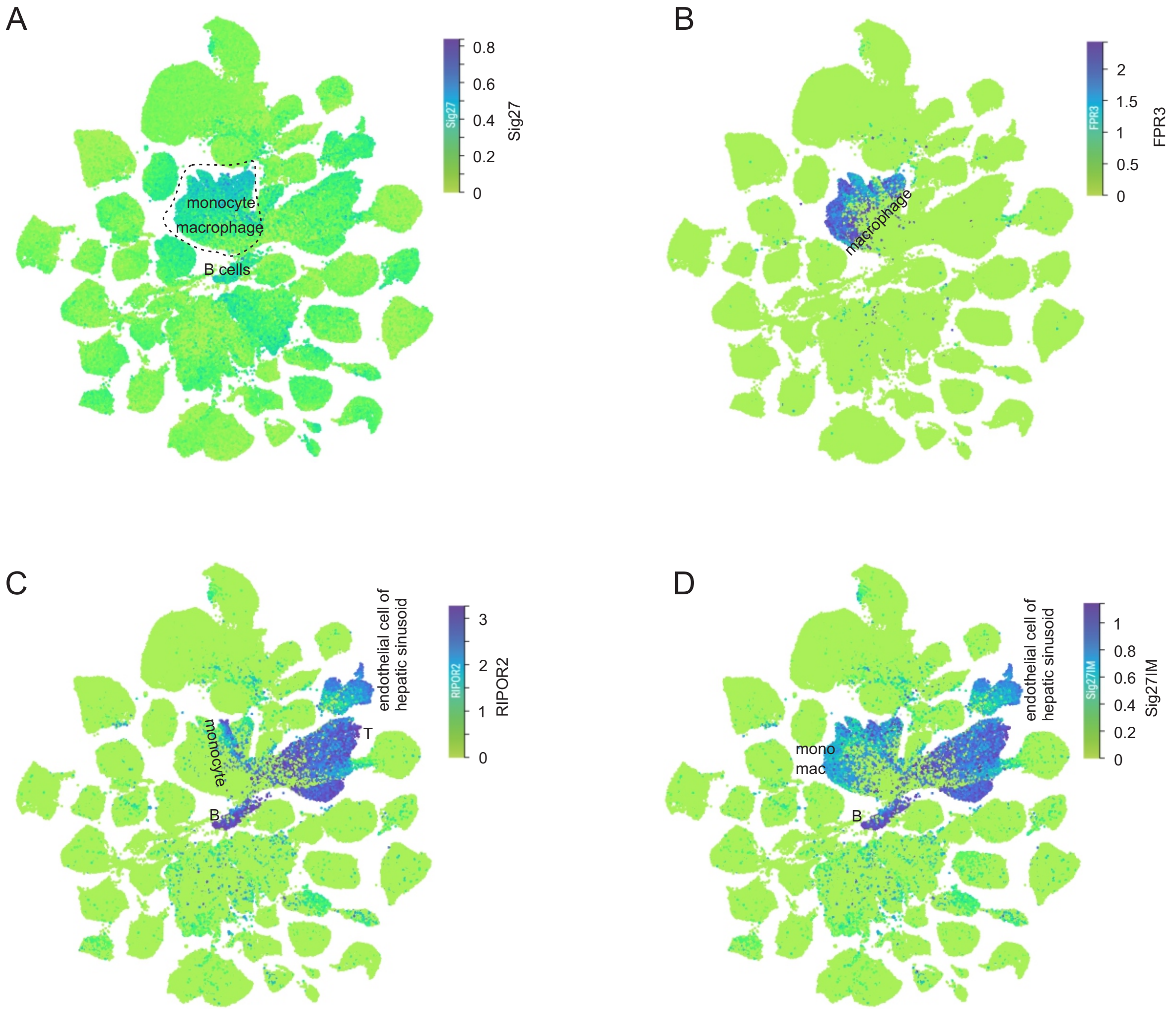

Supplementary Fig. 5. Expression of Sig27 and its component genes in metastatic BC. Analyses were performed using using a single nuclear RNA-seq dataset derived from 60 patients with metastatic BC within the CZ CELLxGENE Discovery website.

Supplementary Figure S6

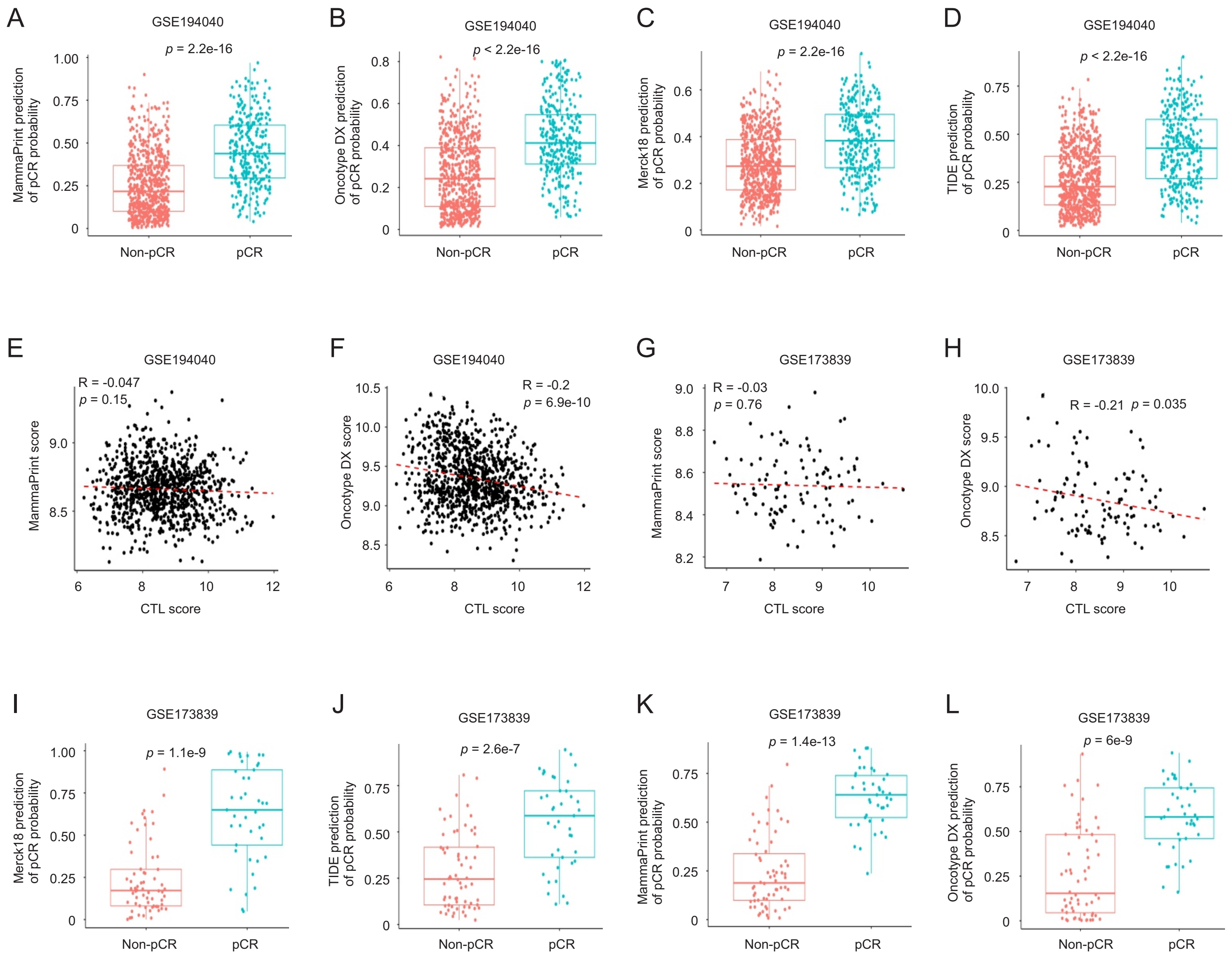

Supplementary Fig. 6. Prediction of pCR. A-D. Predicting the probability of pCR in BCs treated with an array of neoadjuvant regimes in the GSE194040 dataset by logistic modeling of the indicated gene sets. E-H. Pearson correlations of MammaPrint and Oncotype DX with CTL in the indicated datasets. I-L. Predicting the probability of pCR in the GSE173839 dataset by logistic modeling of the indicated gene sets.

Supplementary Figure S7

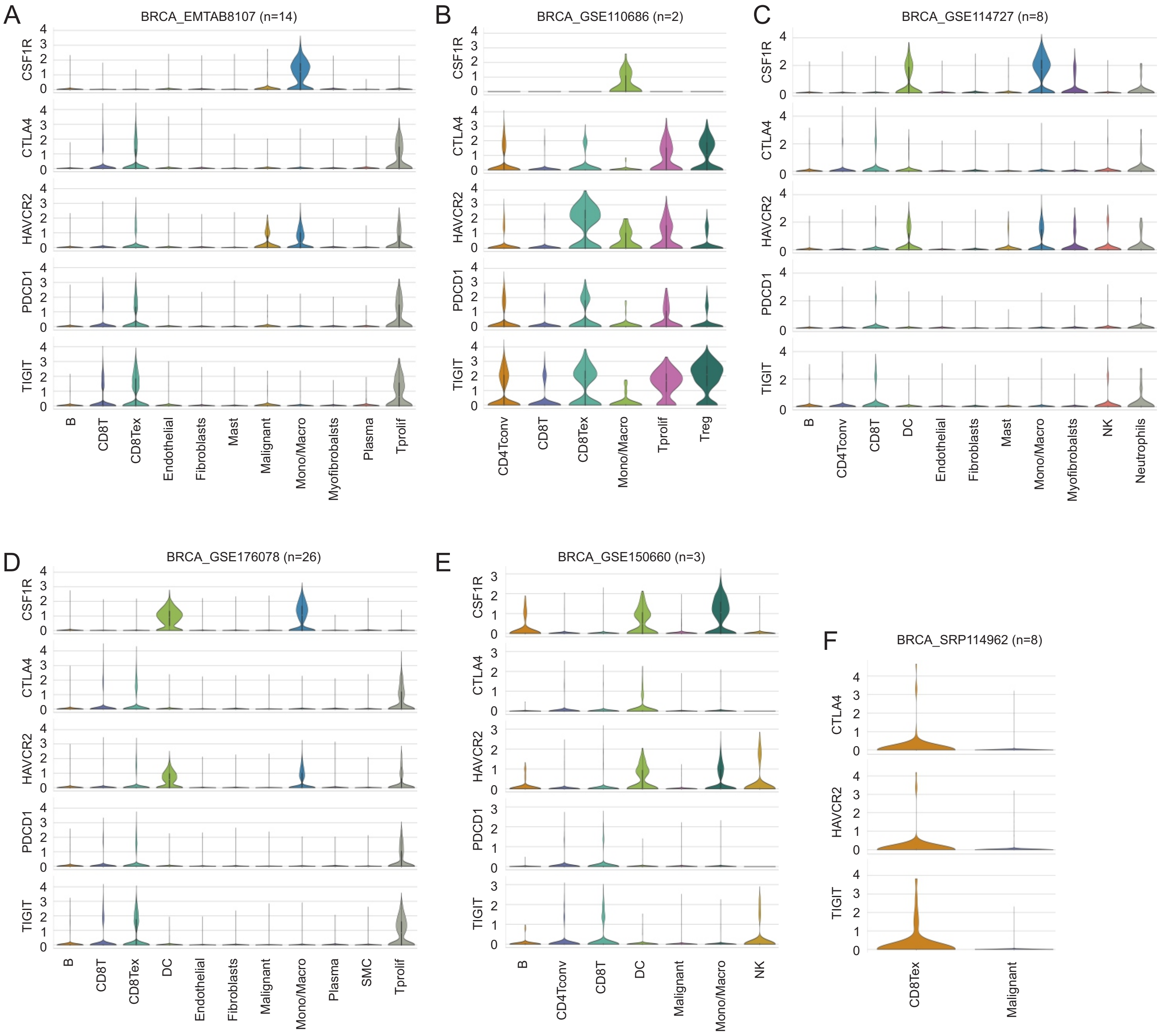

Supplementary Fig. 7. Expression of the indicated ICs in single cell populations of BC. Analyses were performed using the indicated scRNA-seq datasets within the TISCH2 platform.

Supplementary Figure S8

A

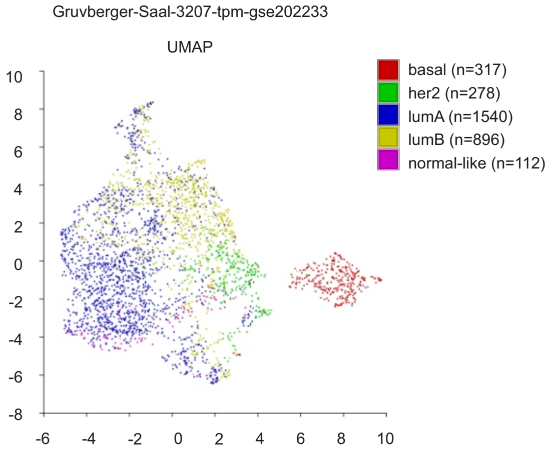

B

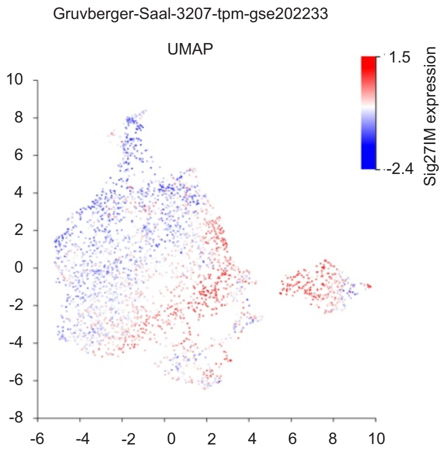

C

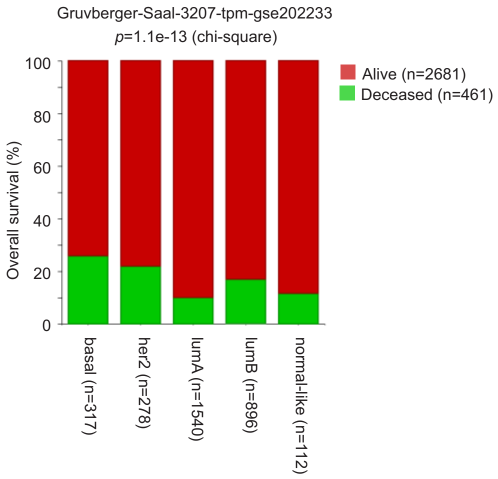

Supplementary Fig. 8. Enrichment of Sig27IM expression in basal-like BC. A. UMAP for the clusters of intrinsic BC subtypes. B. Expression of Sig27IM in intrinsic subtype of BCs. C. Fatality rate of intrinsic BC subtypes.

Supplementary Figure S9

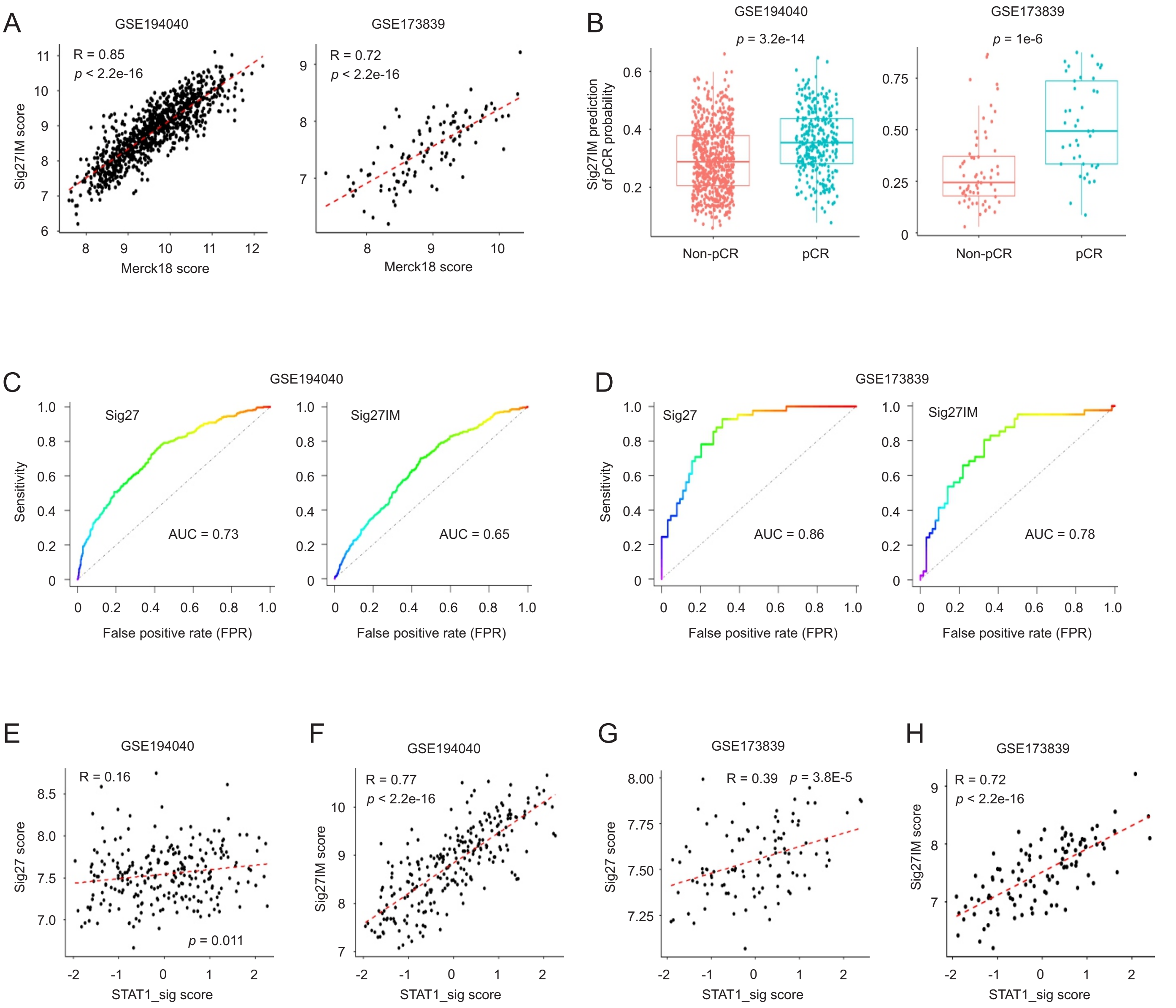

Supplementary Fig. 9. Analysis of pCR in GSE173839 and GSE194040 datasets. A. Pearson correlations of Sig27IM with Merck18. B. Prediction of pCR in the indicated datasets by logistic modeling of Sig27IM. C, D. ROC-AUC curves testing Sig27- and Sig27IM-mediated predicting pCR. E-H. Pearson correlation of Sig27 and Sig27IM with STAT1_sig in tumors treated with PD1 or PDL1 inhibitor in the indicated datasets.
